# Supplementary material for: AI-based discovery and cryoEM structural elucidation of a KATP channel pharmacochaperone
Source: eLife. 2025 Mar 26;13:RP103159. doi: 10.7554/eLife.103159 (PMC11942174; doi:10.7554/eLife.103159)
Supplement: Figure 1—figure supplement 1—source data 1. [file elife-103159-fig1-figsupp1-data1.zip › Figure 1-figure supplement 1._Source Data 1/Figure 1-figure supplement 1._Source Data1.pdf]

C1-10

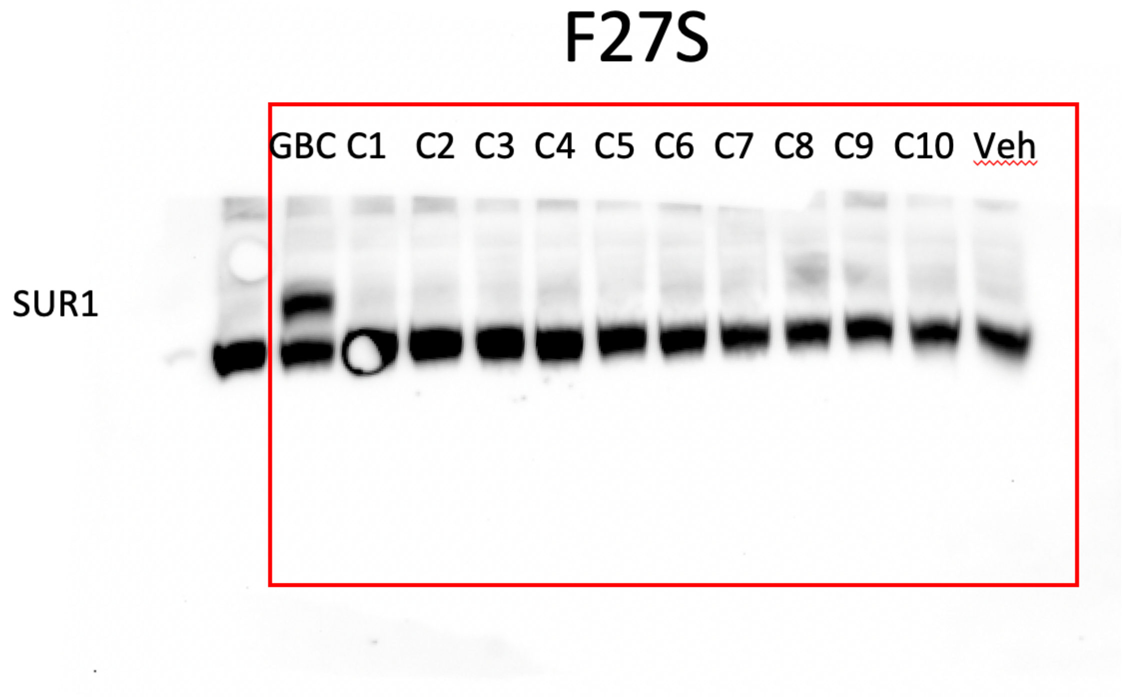
F27S
GBC C1 C2 C3 C4 C5 C6 C7 C8 C9 C10 Veh
SUR1

C11-20

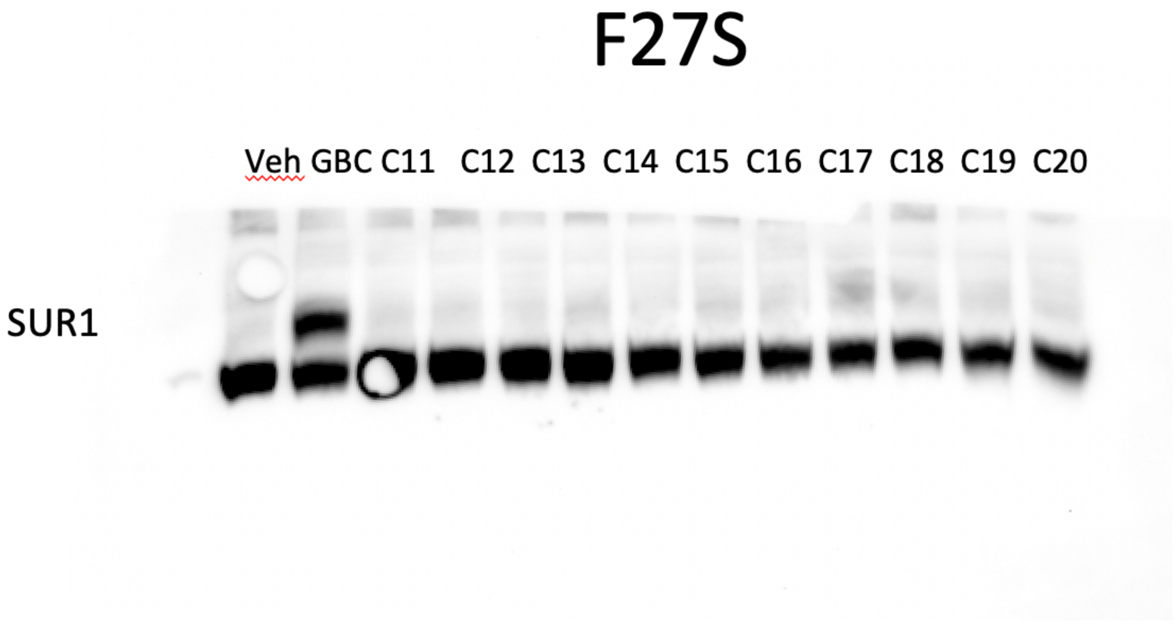
F27S
Veh GBC C11 C12 C13 C14 C15 C16 C17 C18 C19 C20
SUR1

C21-30

F27S

SUR1

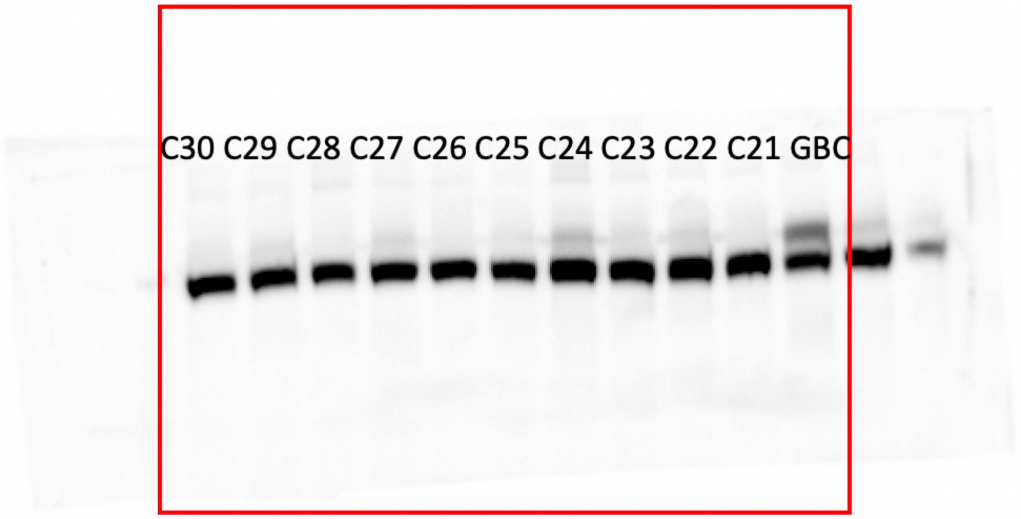

This image was horizontally flipped in Figure 1-figure supplement 1

C31- 40

F27S

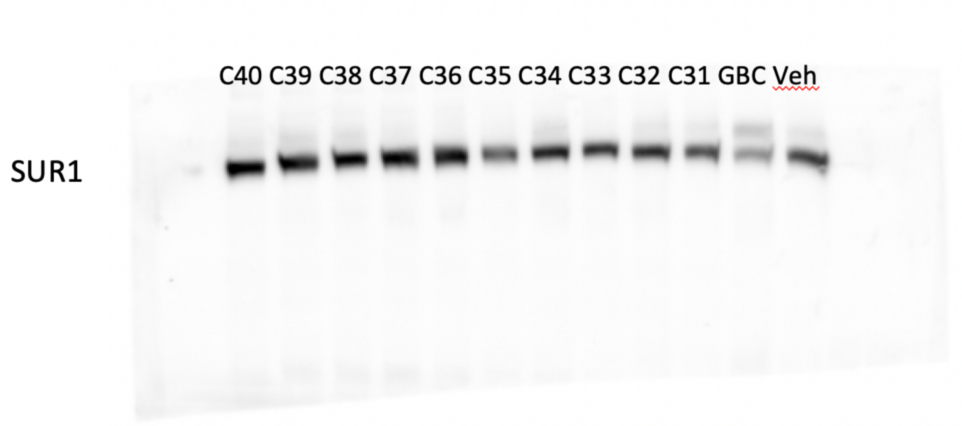

This image was horizontally flipped in Figure 1-figure supplement 1

C41-48

F27S

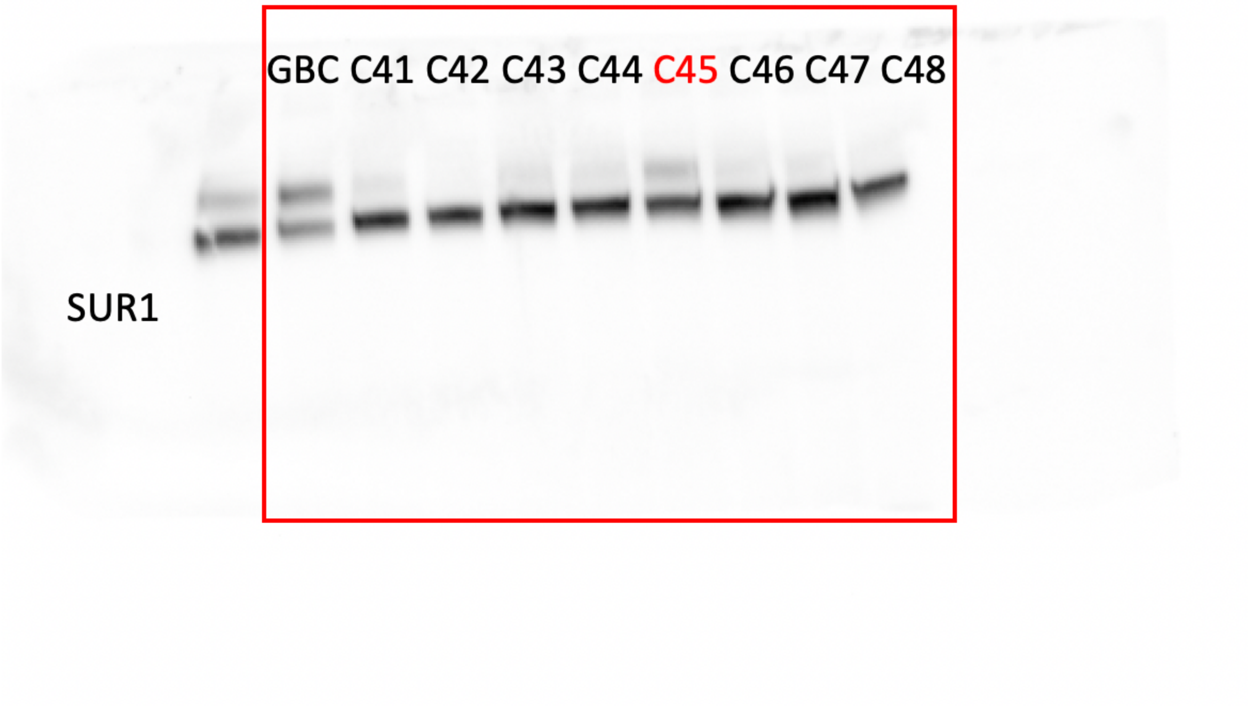

C49-59

F27S

C59 C58 C57 C56 C55 C54 C53 C52 C51 C50 C49 GBC Veh

SUR1

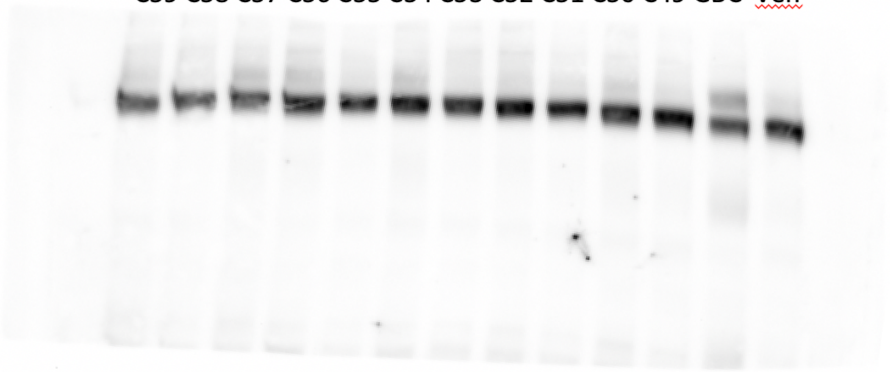

This image was horizontally flipped in Figure 1-figure supplement 1

C60-70

F27S

C70 C69 C68 C67 C66 C65 C64 C63 C62 C61 C60 GBC Veh

SUR1

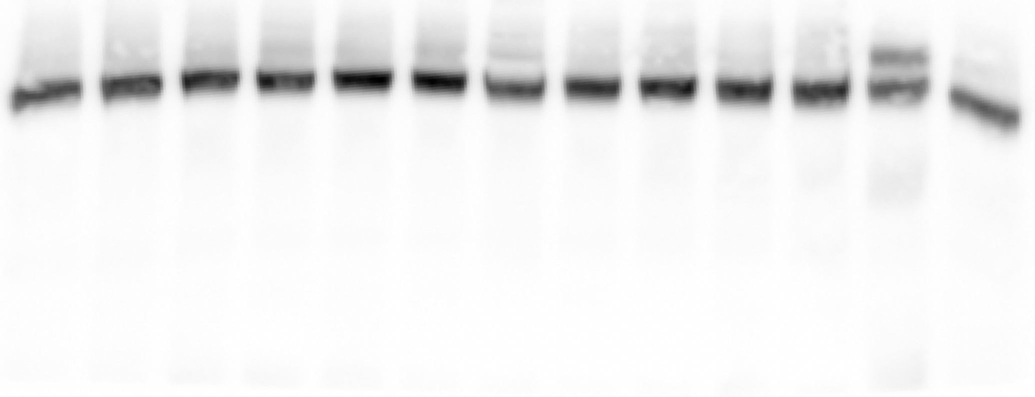

This image was horizontally flipped in Figure 1-figure supplement 1

C71-80

F27S

SUR1

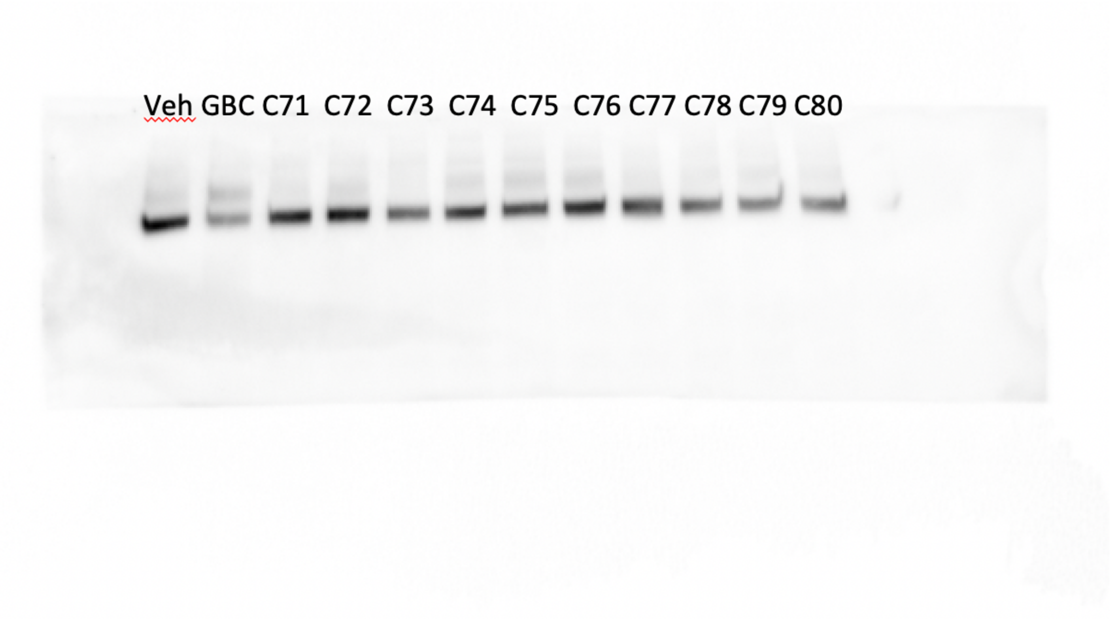

C81-92

F27S

C92 C91 C90 C89 C88C87 C86 C85 C84 C83 C82 C81 Veh

SUR1

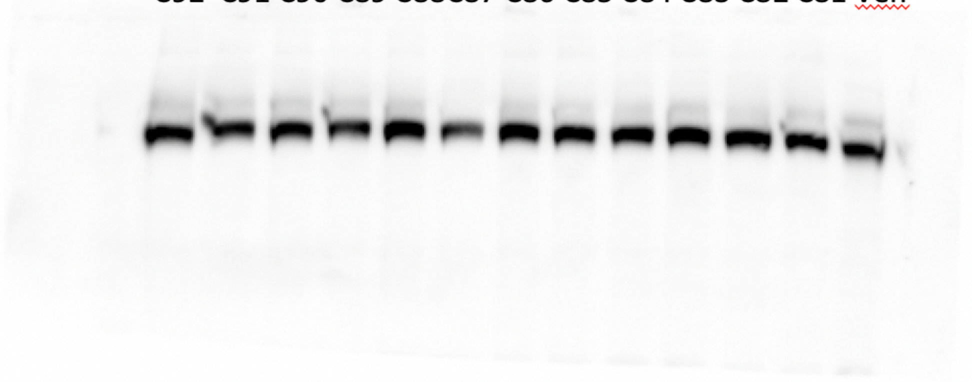

This image was horizontally flipped in Figure 1-figure supplement 1

C93-96

F27S

C96 C95 C94 C93 GBC Veh

SUR1

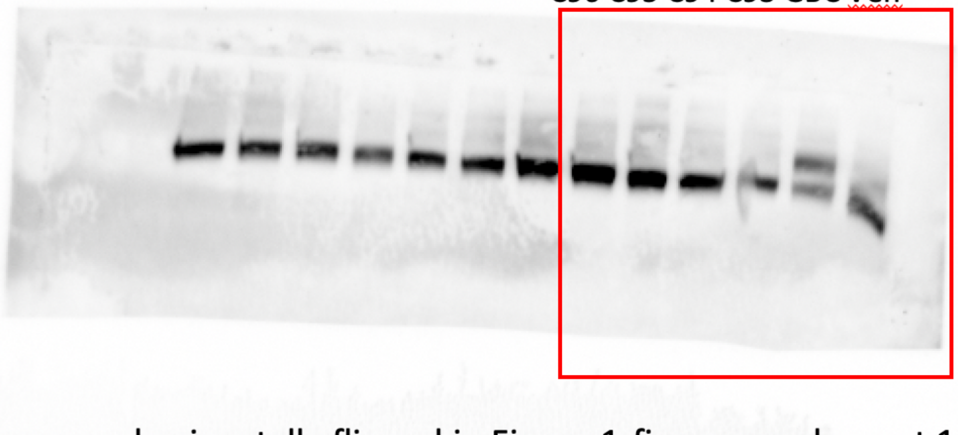

This image was horizontally flipped in Figure 1-figure supplement 1

Figure 1-figure supplement 1, Source Data 1. Original membrane images corresponding to the screening results of Figure 1-figure supplement 1**.**, panel B. The red boxes indicate the specific areas shown in the figure. Please note that some of the images were horizontally flipped (as indicated below the image) in Figure 1-figure supplement 1**.**, panel B. Bands outside these boxes are not included in the figure.
