# Supplementary figures and images for: AI-based discovery and cryoEM structural elucidation of a KATP channel pharmacochaperone
Source: eLife. 2025 Mar 26;13:RP103159. doi: 10.7554/eLife.103159 (PMC11942174; doi:10.7554/eLife.103159)

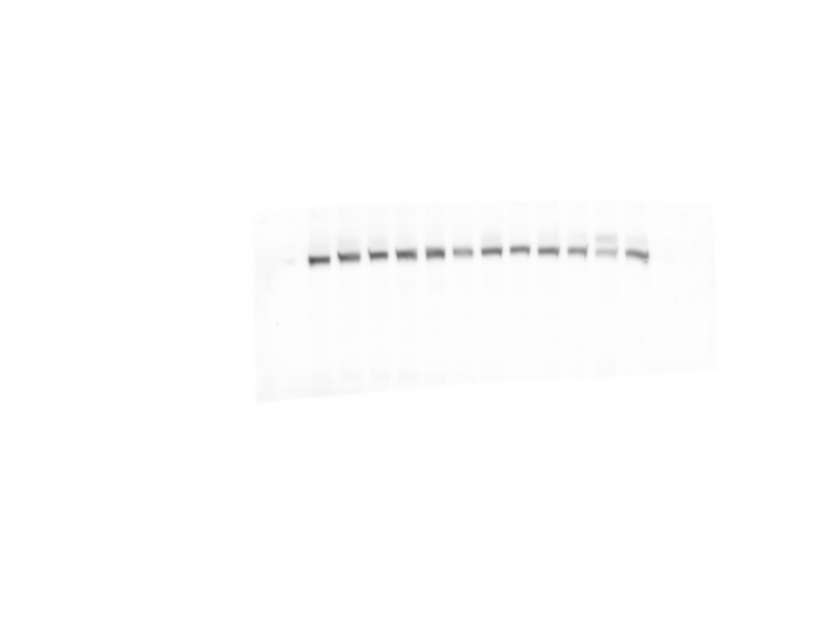

Supplement: Figure 1—figure supplement 1—source data 2. [file elife-103159-fig1-figsupp1-data2.zip › Figure 1-figure supplement 1._Source Data 2/SUR1 F27S-GBC-C31-40 flipped.png]

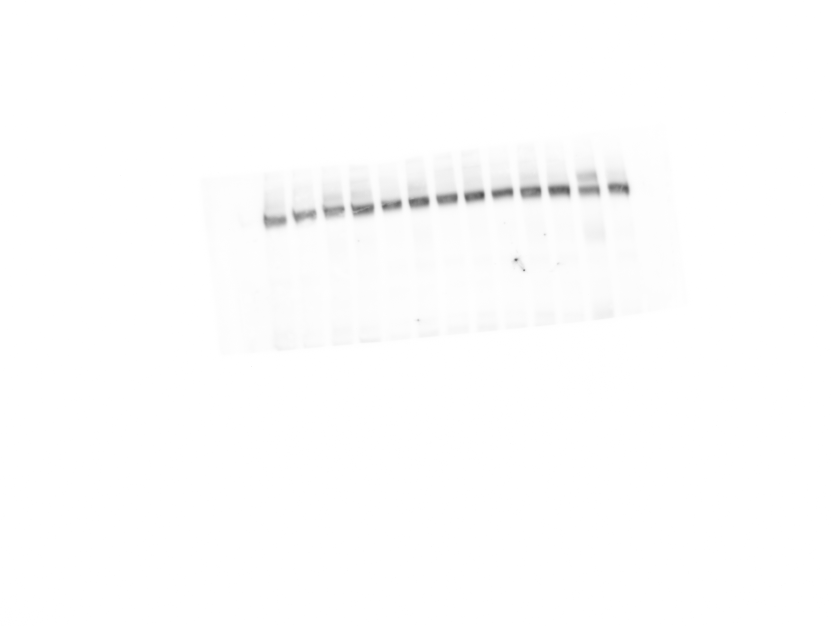

Supplement: Figure 1—figure supplement 1—source data 2. [file elife-103159-fig1-figsupp1-data2.zip › Figure 1-figure supplement 1._Source Data 2/SUR1 F27S-GBC-C49-59 flipped.png]

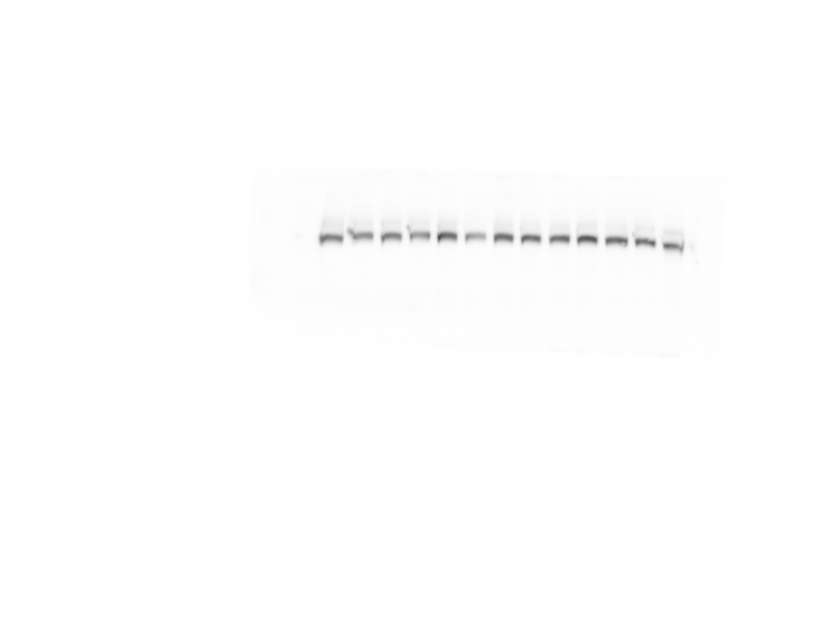

Supplement: Figure 1—figure supplement 1—source data 2. [file elife-103159-fig1-figsupp1-data2.zip › Figure 1-figure supplement 1._Source Data 2/SUR1 F27S-C81-92 flipped.png]

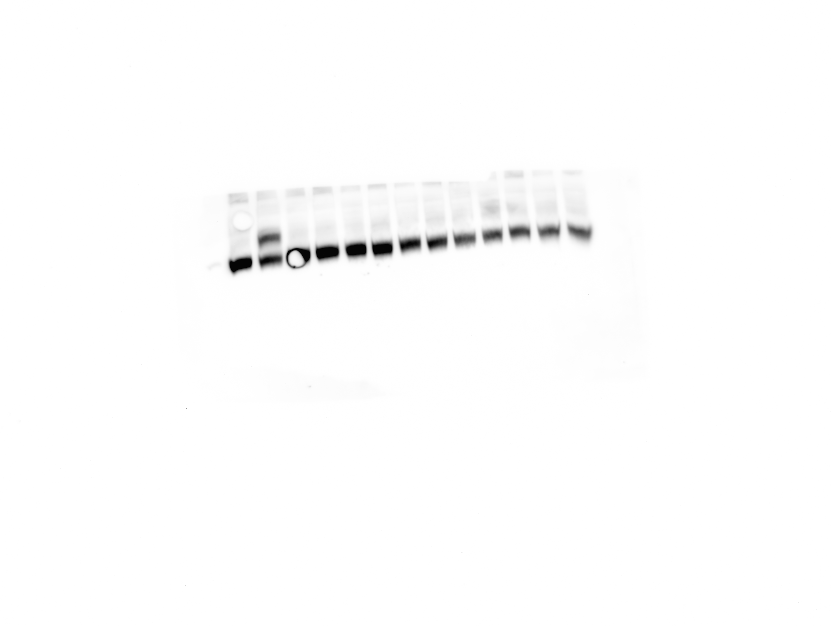

Supplement: Figure 1—figure supplement 1—source data 2. [file elife-103159-fig1-figsupp1-data2.zip › Figure 1-figure supplement 1._Source Data 2/SUR1 F27S-GBC-C1-10.png]

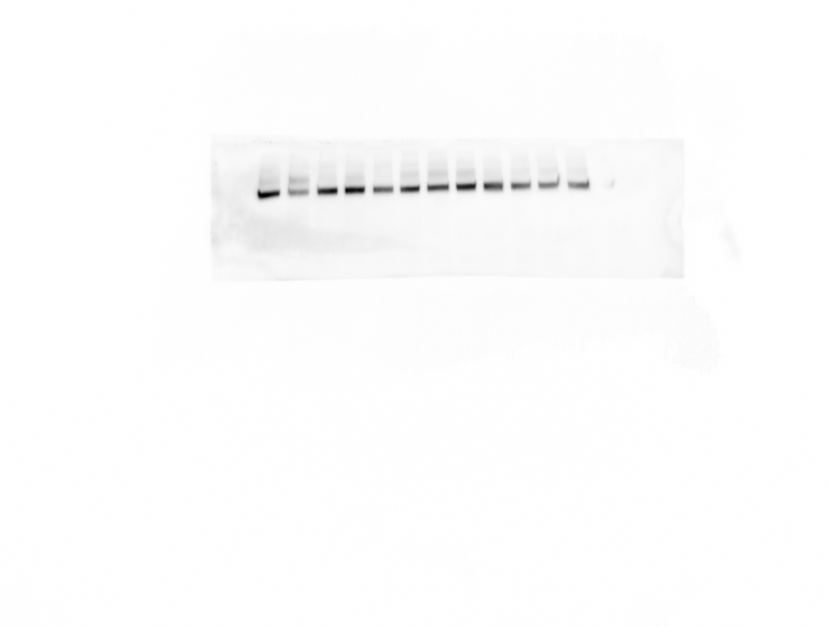

Supplement: Figure 1—figure supplement 1—source data 2. [file elife-103159-fig1-figsupp1-data2.zip › Figure 1-figure supplement 1._Source Data 2/SUR1 F27S-GBC-C71-80.tif]

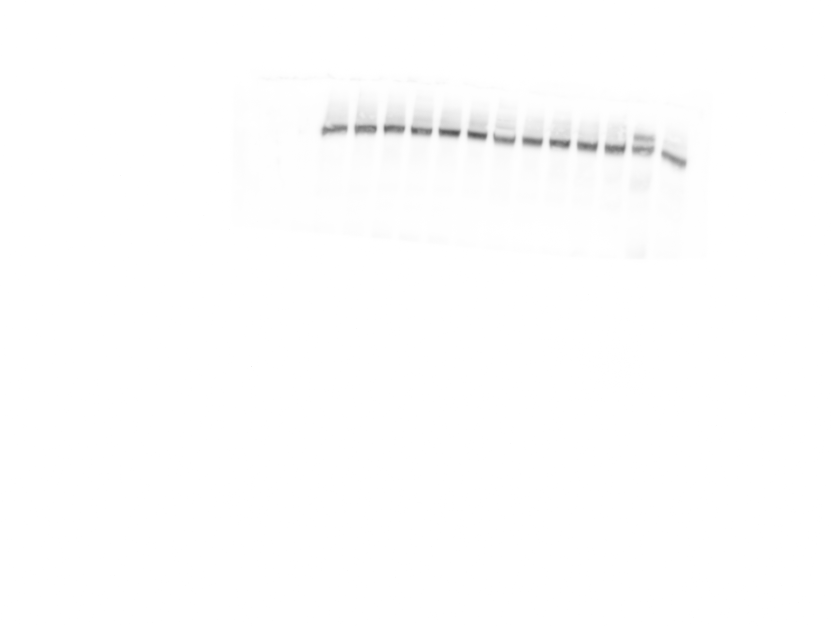

Supplement: Figure 1—figure supplement 1—source data 2. [file elife-103159-fig1-figsupp1-data2.zip › Figure 1-figure supplement 1._Source Data 2/SUR1 F27S-C60-70 flipped.png]

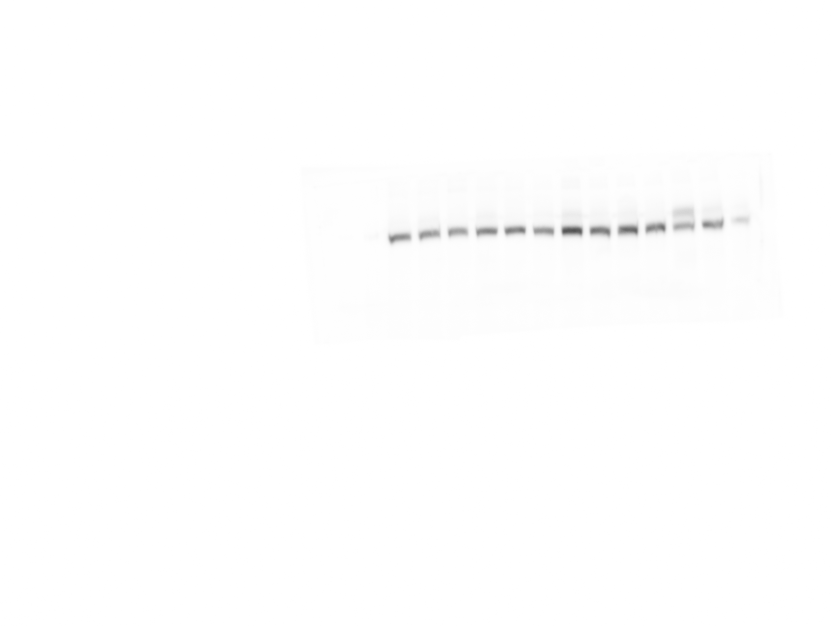

Supplement: Figure 1—figure supplement 1—source data 2. [file elife-103159-fig1-figsupp1-data2.zip › Figure 1-figure supplement 1._Source Data 2/SUR1 F27S-GBC-C21-30 flipped.png]

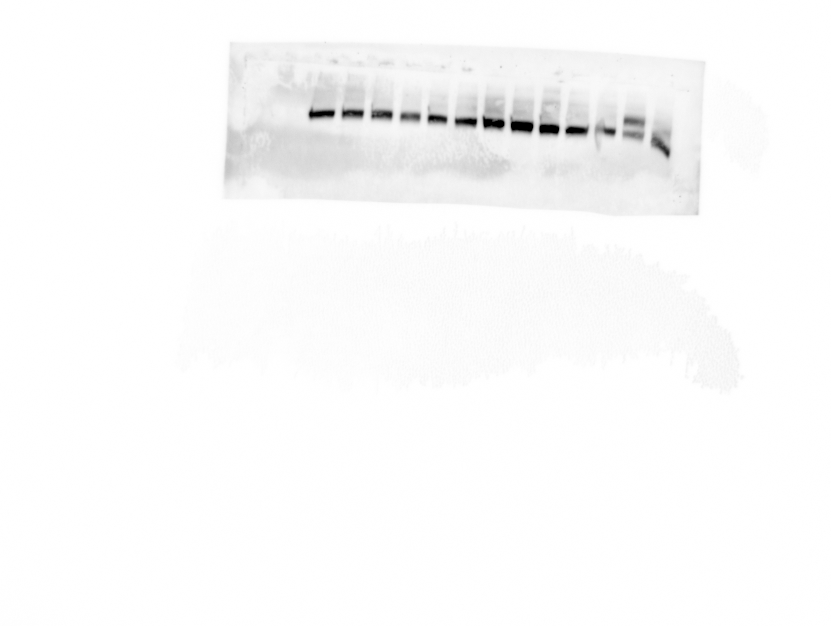

Supplement: Figure 1—figure supplement 1—source data 2. [file elife-103159-fig1-figsupp1-data2.zip › Figure 1-figure supplement 1._Source Data 2/SUR1 F27S-GBC-C93-96 flipped.png]

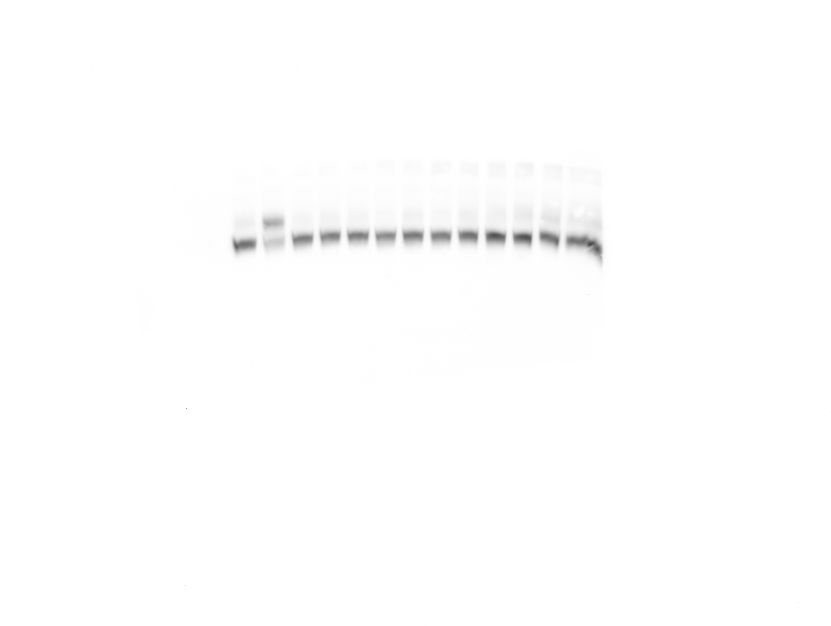

Supplement: Figure 1—figure supplement 1—source data 2. [file elife-103159-fig1-figsupp1-data2.zip › Figure 1-figure supplement 1._Source Data 2/SUR1 F27S-GBC-C11-20.png]

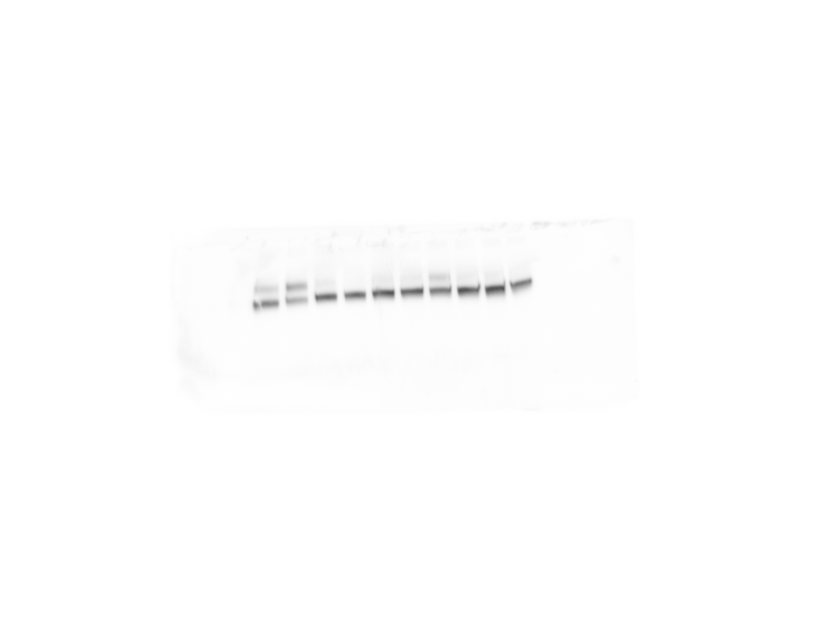

Supplement: Figure 1—figure supplement 1—source data 2. [file elife-103159-fig1-figsupp1-data2.zip › Figure 1-figure supplement 1._Source Data 2/SUR1 F27S-GBC-C41-48.tif]

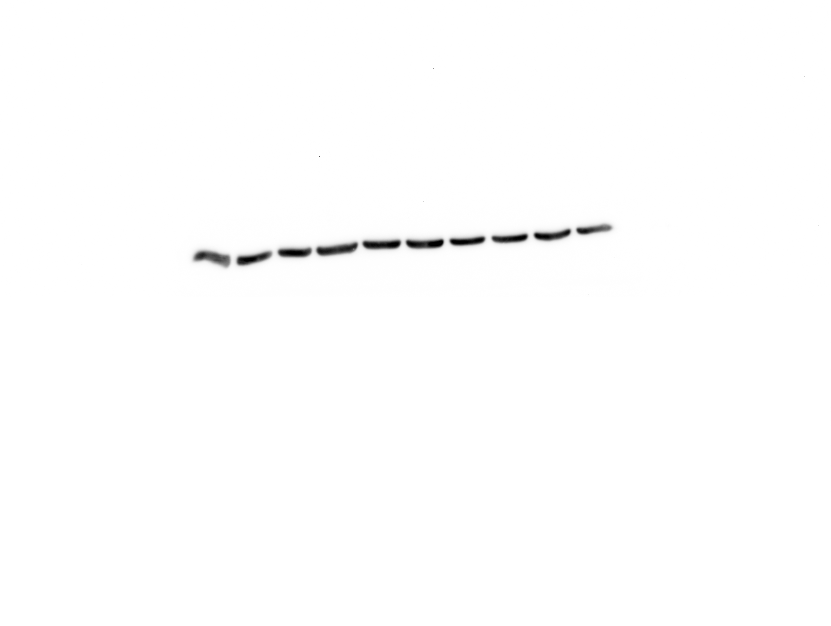

Supplement: Figure 2—source data 2. [file elife-103159-fig2-data2.zip › Figure 2_Source data 2/tubulin AKP on A30T- A116P- V187D.tif]

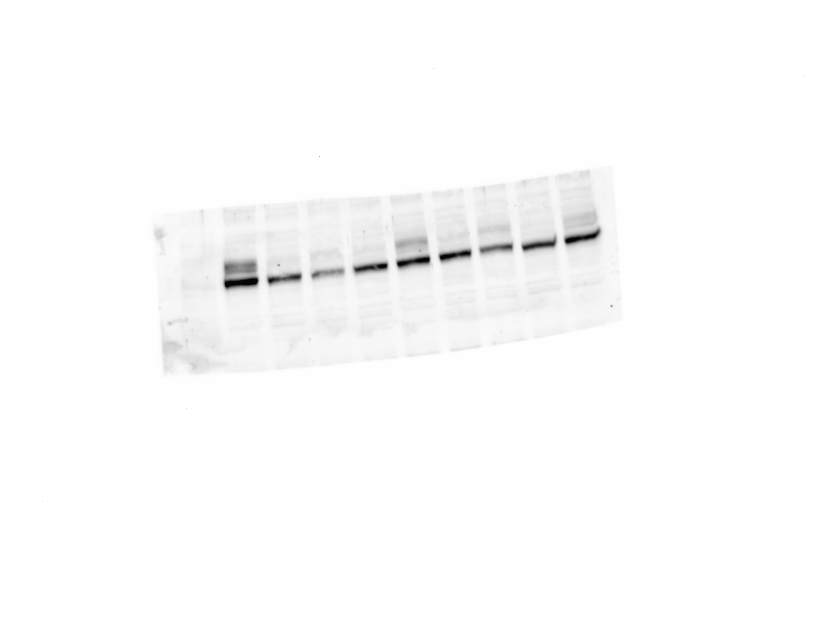

Supplement: Figure 2—source data 2. [file elife-103159-fig2-data2.zip › Figure 2_Source data 2/SUR1 AKP on A30T- A116P- V187D.tif]

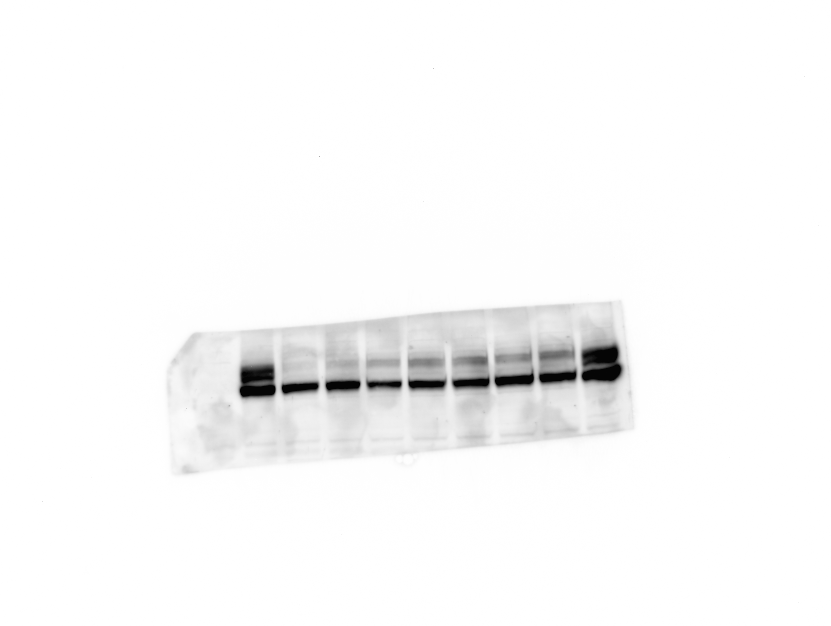

Supplement: Figure 2—source data 2. [file elife-103159-fig2-data2.zip › Figure 2_Source data 2/SUR1 A30T AKP treatment 2.tif]

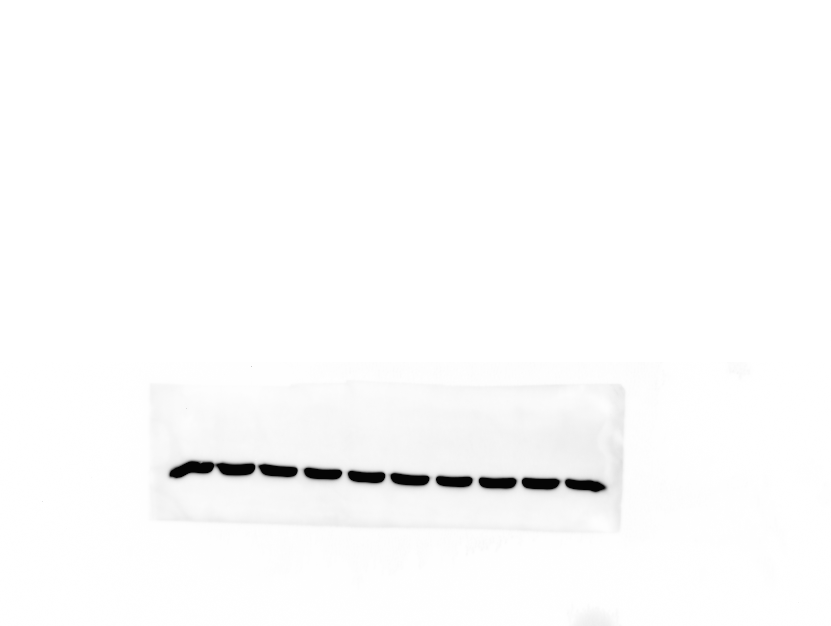

Supplement: Figure 2—source data 2. [file elife-103159-fig2-data2.zip › Figure 2_Source data 2/Tubulin A30T AKP treatment 2.tif]

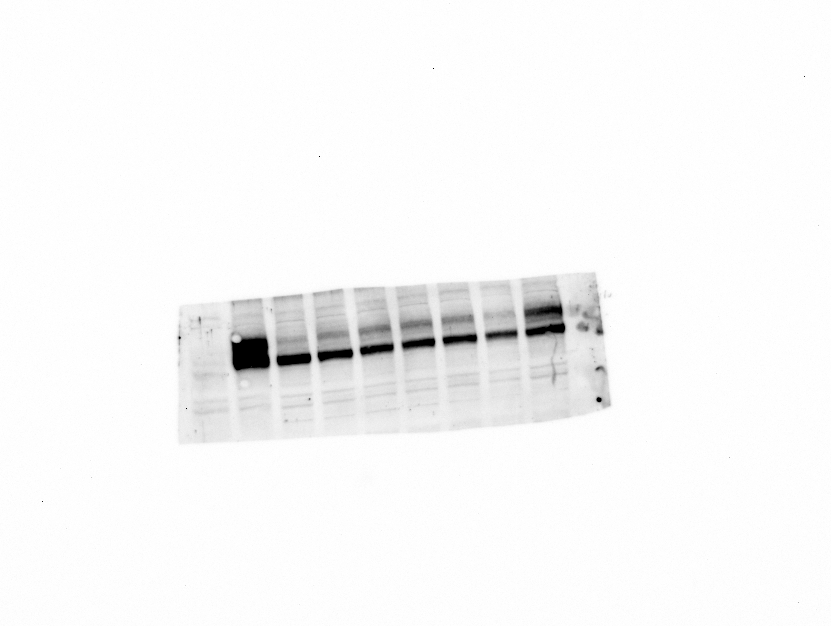

Supplement: Figure 2—figure supplement 2—source data 2. [file elife-103159-fig2-figsupp2-data2.zip › Figure 2-figure supplement 2- Source data-2/SUR1 for human A30T- AKP.tif]

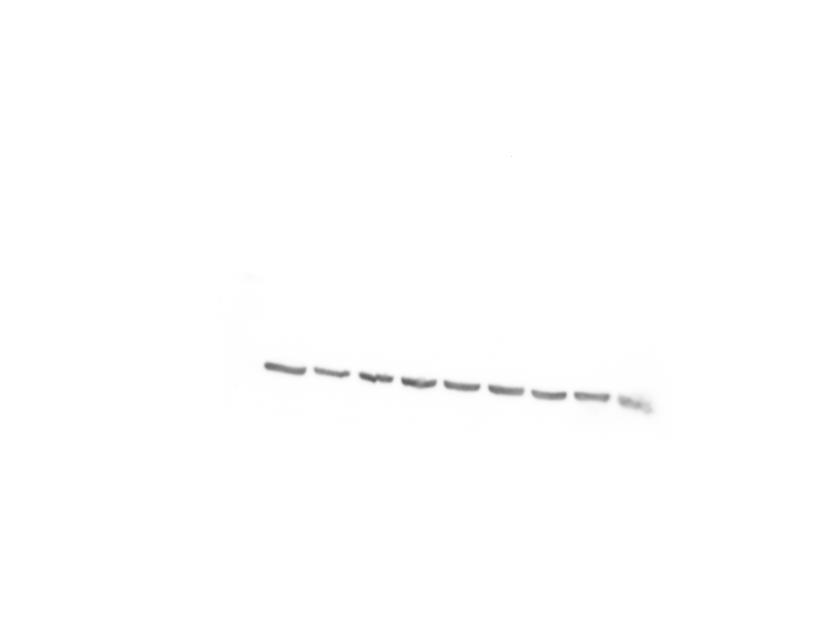

Supplement: Figure 2—figure supplement 2—source data 2. [file elife-103159-fig2-figsupp2-data2.zip › Figure 2-figure supplement 2- Source data-2/tubulin for human A30T- AKP.tif]
