# Supplementary material for: AI-based discovery and cryoEM structural elucidation of a KATP channel pharmacochaperone
Source: eLife. 2025 Mar 26;13:RP103159. doi: 10.7554/eLife.103159 (PMC11942174; doi:10.7554/eLife.103159)
Supplement: Figure 2—source data 1. [file elife-103159-fig2-data1.zip › Figure 2_Source Data 1/Figure 2 _Source Data 1.pdf]

Figure 2 A

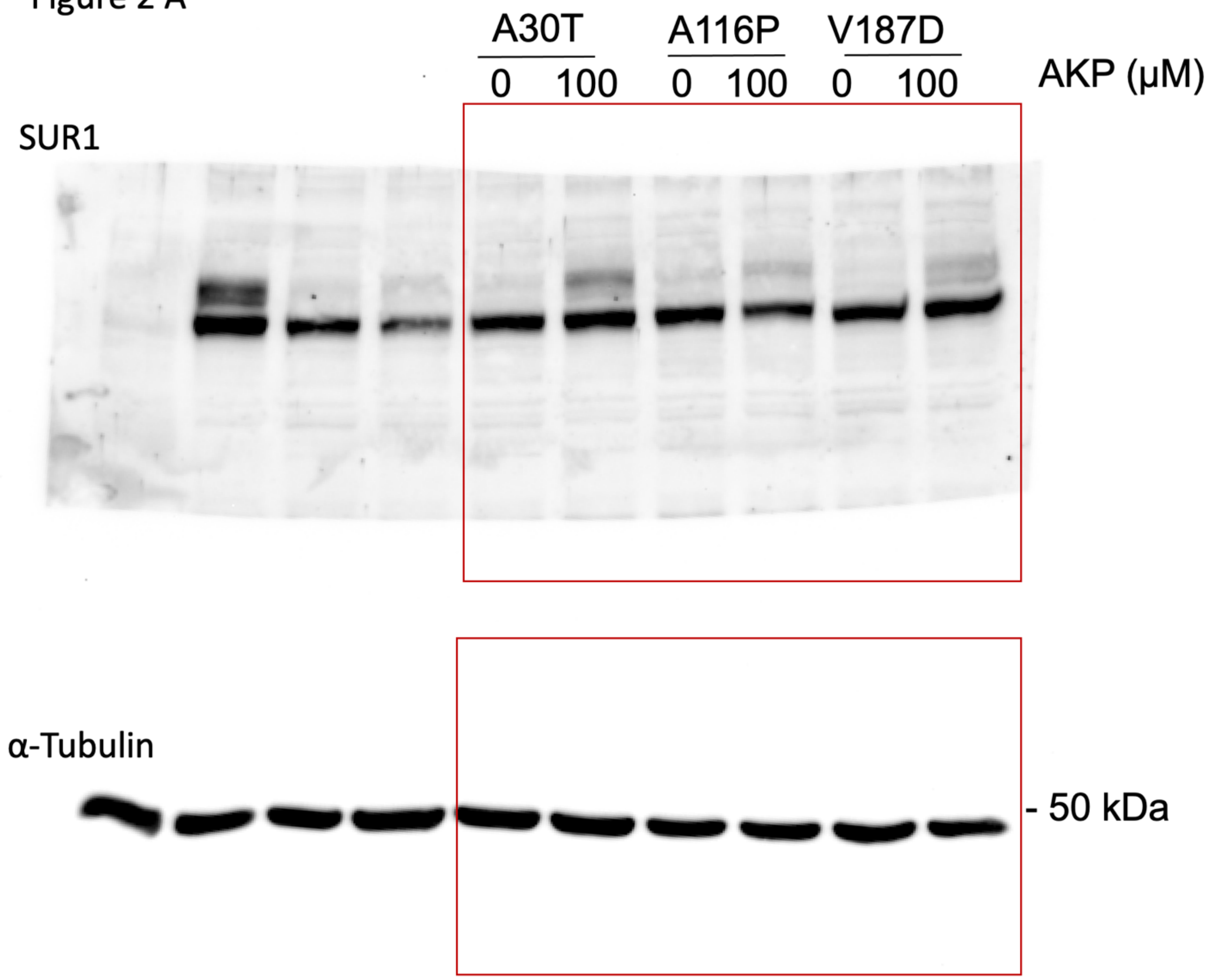

Figure 2 B

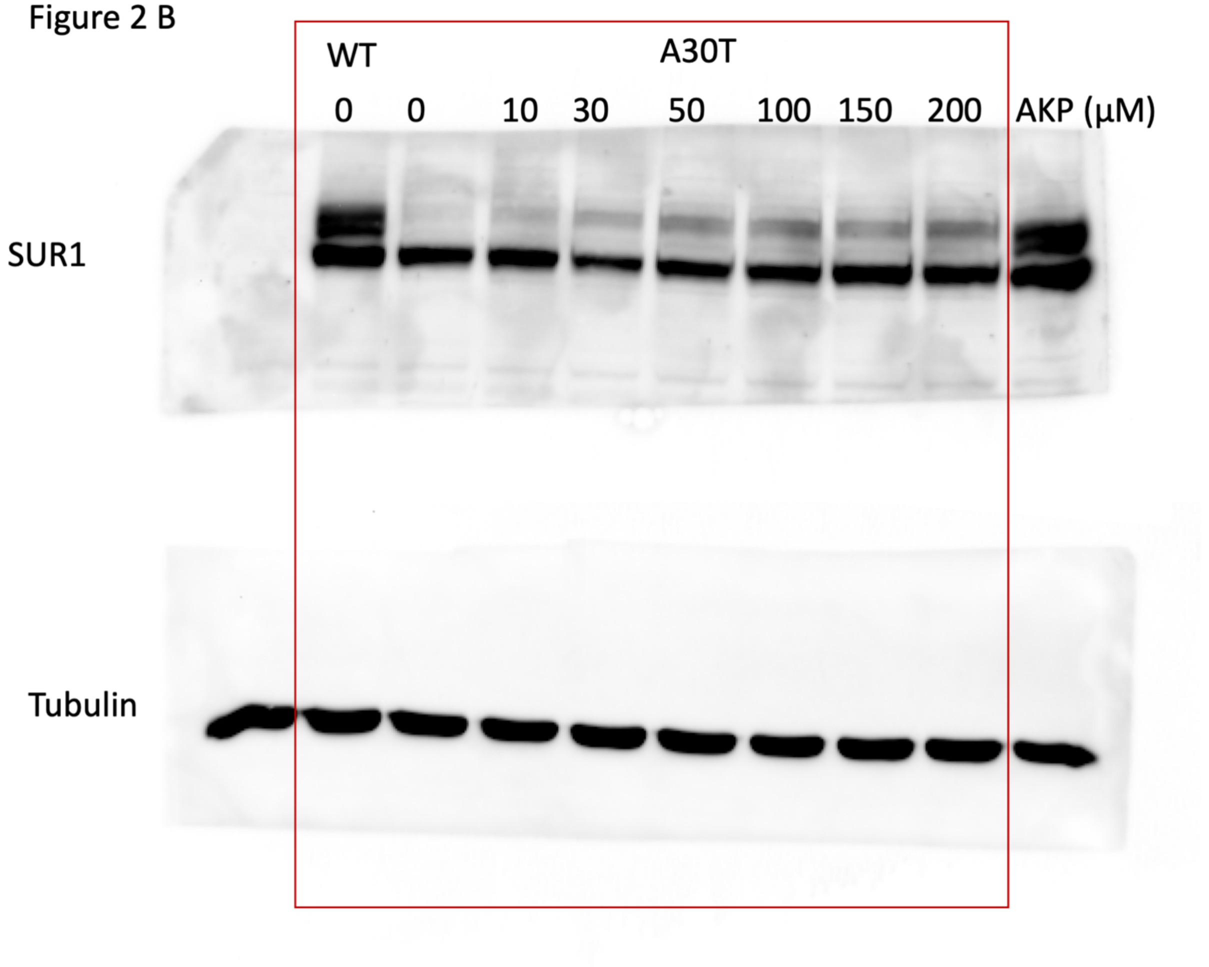

Figure 2, Source Data 1. Original membrane images corresponding to Figure 2, panels A and B. The red boxes highlight the specific areas represented in Figure 2. Bands outside these boxes are not included in the figure.
