# Supplementary material for: AI-based discovery and cryoEM structural elucidation of a KATP channel pharmacochaperone
Source: eLife. 2025 Mar 26;13:RP103159. doi: 10.7554/eLife.103159 (PMC11942174; doi:10.7554/eLife.103159)
Supplement: Figure 2—figure supplement 2—source data 1. [file elife-103159-fig2-figsupp2-data1.zip › Figure 2-figure supplement 2- Source data-1/Figure 2-figure supplement 2_Source Data 1.pdf]

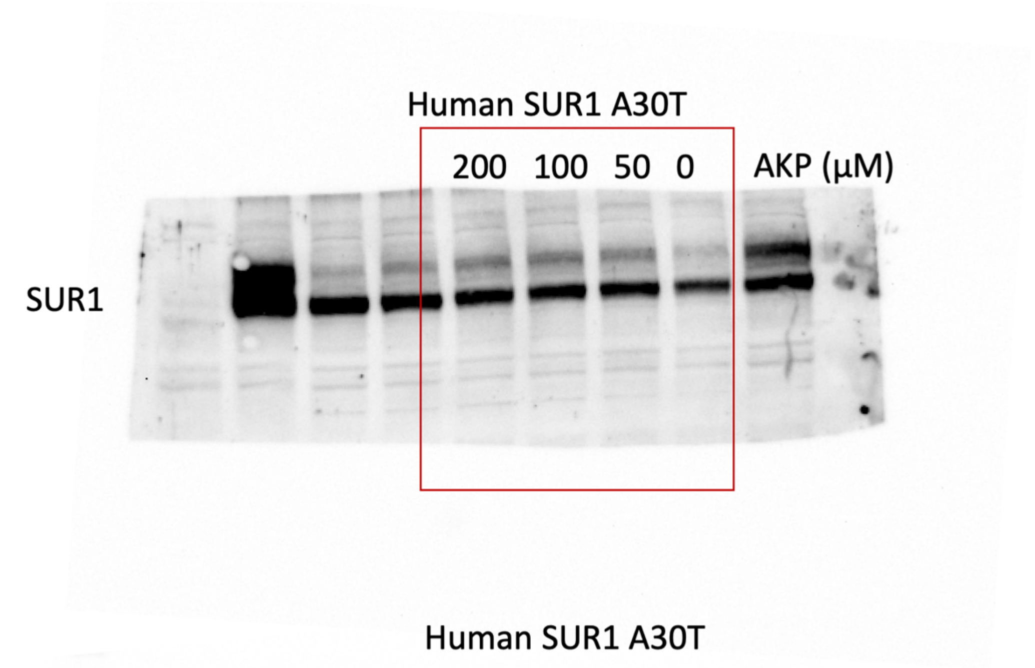


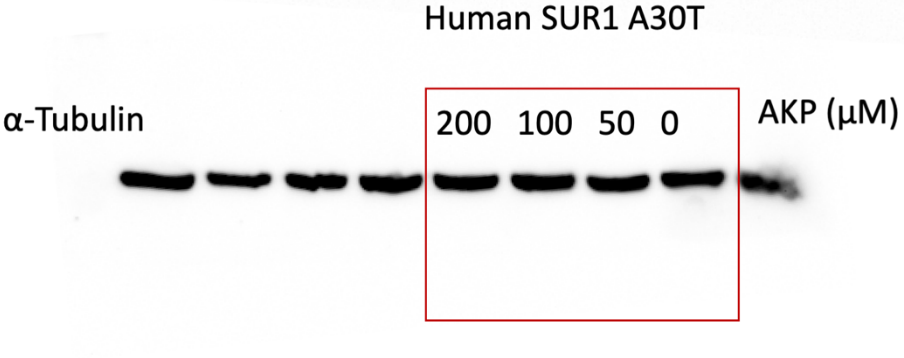


**Figure 2-figure supplement 2, Source Data 1.** Original membrane images corresponding to Figure 2-figure supplement 2, panel A. The red boxes indicate the specific areas shown in the figure. Please note that the image was horizontally flipped in Figure 2-figure supplement 2, panel A. Bands outside these boxes are not included in the figure.
