# Supplementary material for: AI-based discovery and cryoEM structural elucidation of a KATP channel pharmacochaperone
Source: eLife. 2025 Mar 26;13:RP103159. doi: 10.7554/eLife.103159 (PMC11942174; doi:10.7554/eLife.103159)
Supplement: Supplementary file 1. [file elife-103159-supp1.docx]

**Supplementary Table 1.** **List of predicted binders that were subjected to biochemical and functional testing***

| **Compound (C) Number** | **ID** | **Formula** | **MW** | **Purity** | **Smile** |  |  |  |
| --- | --- | --- | --- | --- | --- | --- | --- | --- |
| C1 | Z278092326 | C22H26N2O3S | 398.518 | 95 | CC=1C(C)=C(C)C(=C(C)C1C)S(=O)(=O)N(CC2=CC=CO2)CC=3C=CC=NC3 |  |  |  |
| C2 | Z112630850 | C20H19N5O2S | 393.462 | 100 | CC(SC1=NNC(=O)N1CC=2C=CC=CC2)C3=NN=C(O3)C=4C=CC=C(C)C4 |  |  |  |
| C3 | Z92024344 | C22H18N4O3 | 386.403 | 100 | OC(C(=O)OCC1=NN=NN1C=2C=CC=CC2)(C=3C=CC=CC3)C=4C=CC=CC4 |  |  |  |
| C4 | Z595640450 | C22H22N2O4 | 378.421 | 98 | O=C(NCC1CC=2C=CC=CC2O1)NC=3C=CC=C(COCC4=CC=CO4)C3 |  |  |  |
| C5 | Z299683904 | C19H15N3O2S | 349.406 | 95 | O=S(=O)(NC=1C=CC=C2C=NNC12)C=3C=CC=CC3C=4C=CC=CC4 |  |  |  |
| C6 | Z1066092860 | C19H19N3O3S | 369.437 | 93 | COC=1C=CC=CC1S(=O)(=O)NC2=C3CCCC3=NN2C=4C=CC=CC4 |  |  |  |
| C7 | Z1686879312 | C15H21N5O2S | 335.424 | 96 | CC(C)C=1C=C(NS(=O)(=O)CCC2CC2)N(N1)C=3N=CC=CN3 |  |  |  |
| C8 | Z1643193385 | C18H20N2O2S | 328.428 | 96 | OC1(CNC(=O)N(C=2C=CC=CC2)C=3C=CC=CC3)CCSC1 |  |  |  |
| C9 | Z1675091485 | C17H14N2O3S | 326.369 | 95 | CC(=O)C1=CSC(=C1)C(=O)NC=2C(C)=NOC2C=3C=CC=CC3 |  |  |  |
| C10 | Z1731682842 | C21H19N3O2 | 345.394 | 100 | OC1CC=2C=CC=CC2C1NC(=O)NC=3C=CC=CC3C=4C=CN=CC4 |  |  |  |
| C11 | Z1833488549 | C15H12Cl2N4O2S | 383.252 | 100 | ClC=1C=CC=C(Cl)C1CS(=O)(=O)NC2=CNN=C2C=3C=CC=CN3 |  |  |  |
| C12 | Z1542063934 | C17H15F3N4O2S | 396.386 | 100 | CC=1C=C(NS(=O)(=O)CC=2C=CC=CC2)N(N1)C=3C=CC(=CN3)C(F)(F)F |  |  |  |
| C13 | Z1138487193 | C19H16N4O2 | 332.355 | 100 | O=C(OCCC1=CNC=2C=CC=CC12)C=3C=NN(N3)C=4C=CC=CC4 |  |  |  |
| C14 | Z1838052631 | C21H23N3O3 | 365.425 | 95 | CC(NC(=O)NCC=1C=CC(=CC1)C2CC2)C=3C=CC=4OCC(=O)NC4C3 |  |  |  |
| C15 | Z1160819972 | C16H16ClN5O2 | 345.783 | 90 | OC(COCC=1C=CC(Cl)=CC1)CN2N=NN=C2C=3C=CC=CN3 |  |  |  |
| C16 | Z1955643944 | C20H23N3O3S | 385.479 | 100 | CN1N=CC=C1C=2C=CC=CC2OCCN(CC=3C=CC=CC3)S(=O)(=O)C |  |  |  |
| C17 | Z1393172784 | C19H15ClN4O2S | 398.866 | 91 | ClC=1C=CC(=C2C=CC=CC12)S(=O)(=O)NC3=NN=CN3CC=4C=CC=CC4 |  |  |  |
| C18 | Z56788102 | C20H16N2O2 | 316.353 | 100 | O=C(NC=1C=CC=CC1NC(=O)C=2C=CC=CC2)C=3C=CC=CC3 |  |  |  |
| C19 | Z997821168 | C20H17N3O3S | 379.432 | 100 | CN(C=1C=CC=CC1OC=2C=CC=CC2)S(=O)(=O)C3=CNC=4N=CC=CC34 |  |  |  |
| C20 | Z1101903992 | C19H14F2N2O2 | 340.323 | 100 | CC1=CC=C(C(=O)NC=2C=CC(F)=CC2C=3C=CC(F)=CC3)C(=O)N1 |  |  |  |
| C21 | Z425545360 | C18H21N5O2S | 371.456 | 95 | CC=1C(C)=C(C)C(=C(C)C1C)S(=O)(=O)NC=2C=CC=CC2N3C=NN=N3 |  |  |  |
| C22 | Z806302862 | C20H19N3O3 | 349.383 | 92 | CC=1C=C(C=CC1N2CCNC2=O)C(=O)NCC3=CC=4C=CC=CC4O3 |  |  |  |
| C23 | Z1127155602 | C21H20FN3O | 349.401 | 92 | CN(CC=1C=CC(=CC1)C(=O)N)C(C=2C=CC(F)=CC2)C=3C=CC=NC3 |  |  |  |
| C24 | Z1607618869 | C23H24N4O2 | 388.462 | 91 | CN1C(=O)CCC=2C=C(NC(=O)C=3C=C(C)N(N3)C=4C=C(C)C=C(C)C4)C=CC12 |  |  |  |
| C25 | Z1781382199 | C15H12N2OS2 | 300.398 | 96 | CC1=CC(=CS1)C=2N=CC=CC2NC(=O)C3=CC=CS3 |  |  |  |
| C26 | Z2234686034 | C20H19N5O2S | 393.462 | 91 | CN(C)C=1C=CC=C2C(=CC=CC12)S(=O)(=O)NC=3C=CC=CC3C4=NN=CN4 |  |  |  |
| C27 | Z2068224500 | C17H17N3O3S | 343.400 | 90 | O=S(=O)(NC=1C=CC=CC1C2=NC=C3CCCCN23)C=4C=COC4 |  |  |  |
| C28 | Z1997320230 | C21H21N3O2 | 347.410 | 94 | CC(NCC=1C=CC(NC(=O)C=2C=CC=NC2)=CC1)C=3C=CC=C(O)C3 |  |  |  |
| C29 | Z434838868 | C21H19N3O3 | 361.393 | 95 | COC=1C=CC(OC=2N=CC=CC2NC(=O)N3CCC=4C=CC=CC34)=CC1 |  |  |  |
| C30 | Z1317925131 | C23H23N3O2 | 373.447 | 98 | COC=1C=CC(CN(C)CC2=C(N=C3C=CC=CN23)C=4C=CC=CC4)=CC1O |  |  |  |
| C31 | Z1332832275 | C18H16N2O2S | 324.396 | 100 | CC=1C=CC(=CC1)C=2C=CSC2C(=O)NC=3C=NC(O)=CC3C |  |  |  |
| C32 | Z1065893590 | C16H15ClN4O3S | 378.833 | 100 | CC1=NN(C)C(OC=2C=CC=NC2)=C1NS(=O)(=O)C=3C=CC=CC3Cl |  |  |  |
| C33 | Z1430522786 | C15H17F2N5O2S | 369.389 | 99 | CC(C)N1N=CC=2C=C(C=CC12)S(=O)(=O)NC=3C=NN(CC(F)F)C3 |  |  |  |
| C34 | Z751820632 | C16H14N2O2S3 | 362.489 | 91 | O=S(=O)(N1CCC=2SC=CC2C1C3=CC=CS3)C=4C=CC=NC4 |  |  |  |
| C35 | Z857281148 | C21H22N4O3 | 378.424 | 96 | CC=1C=CC=C(C)C1N2N=NN=C2COC(=O)C=3C=C4CCCCC4=CC3O |  |  |  |
| C36 | Z285781140 | C18H20N4OS | 340.442 | 95 | CCC1=CC=2C(=NC=NC2S1)N3CCN(CC3)C=4C=CC(O)=CC4 |  |  |  |
| C37 | Z361864720 | C18H17N3O2S | 339.411 | 95 | O=S(=O)(N(CC=1C=CC=C2C=CC=NC12)C3CC3)C=4C=CC=NC4 |  |  |  |
| C38 | Z1450943637 | C16H16N4O2S2 | 360.453 | 93 | CN1C=C(C=N1)S(=O)(=O)NC=2SC(=NC2C=3C=CC=CC3)C4CC4 |  |  |  |
| C39 | Z1833450474 | C19H21N5O2S | 383.467 | 92 | CN1CCCC=2C=CC(=CC12)S(=O)(=O)NC3=CN(CC=4C=CC=CC4)N=N3 |  |  |  |
| C40 | Z1878392989 | C18H22N4O3 | 342.392 | 96 | O=C1CCCC=2C(OCC3=NN=NN3CC4CCOCC4)=CC=CC12 |  |  |  |
| C41 | Z1187995755 | C19H19FN2O3 | 342.364 | 91 | CC(NC1CCOC1=O)C=2C=CC(NC(=O)C=3C=CC(F)=CC3)=CC2 |  |  |  |
| C42 | Z239150122 | C23H18N2O4 | 386.400 | 100 | O=C(NC=1C=CC=C(OC=2C=CC=CC2)C1)C3=CC=C(CN4C=CC=CC4=O)O3 |  |  |  |
| C43 | Z184527044 | C21H25N3O2 | 351.442 | 100 | CC(=O)NC=1C=CC(NC(=O)C=2C=CC(CN3CCCCC3)=CC2)=CC1 |  |  |  |
| C44 | Z955248736 | C20H20N2O4 | 352.383 | 91 | O=C(NC=1C=CC=CC1C(=O)N2CCOCC2)C=3C=CC=4COCC4C3 |  |  |  |
| C45 | Z1620764636 | C20H25N5O2S | 399.509 | 100 | O=S(=O)(NC1=CC(CC2CCCCC2)=NN1)C=3C=CC=CC3CN4C=CN=C4 |  |  |  |
| C46 | Z827062904 | C13H10F3N5OS | 341.311 | 100 | CC(=O)C=1C=CC=CC1NC2=NN3C(=NN=C3SC2)C(F)(F)F |  |  |  |
| C47 | Z19653453 | C14H9N3OS3 | 331.435 | 100 | O=C1NC(CSC2=NC=3C=CC=CC3S2)=NC=4C=CSC14 |  |  |  |
| C48 | Z1819546701 | C21H20N6O2 | 388.422 | 100 | CC(OC(=O)C1=CN(N=C1C=2C=CC=CC2C)C=3C=CC=CC3)C4=NN=NN4C |  |  |  |
| C49 | Z1836331215 | C21H23N3O3S | 397.490 | 93 | CN1C=C(CNS(=O)(=O)CCC=2C=CC=3OCCC3C2)C(=N1)C=4C=CC=CC4 |  |  |  |
| C50 | Z1071096112 | C16H17BrN2O2 | 349.222 | 100 | COC=1C=CC(Br)=C(CNCC=2C=CC=C(C2)C(=O)N)C1 |  |  |  |
| C51 | Z225706824 | C14H17N3OS2 | 307.434 | 100 | CC=1C=C(SC1C)C(=O)N2CCN(CC2)C3=NC=CS3 |  |  |  |
| C52 | Z202727764 | C20H18ClN3O4 | 399.827 | 99 | CC=1C=CC(OC=2C=CC(Cl)=CC2NC(=O)COC(=O)C=3C=NN(C)C3)=CC1 |  |  |  |
| C53 | Z229456558 | C21H21N5O3 | 391.423 | 92 | O=C(C1CCN(CC1)C2=NN=NN2C=3C=CC=CC3)C=4C=CC=5OCCOC5C4 |  |  |  |
| C54 | Z281793560 | C22H23N3O3 | 377.436 | 100 | CN(C)C(CNC(=O)C=1C=CC(NC(=O)C=2C=CC=CC2)=CC1)C3=CC=CO3 |  |  |  |
| C55 | Z199879632 | C21H21N3O3S | 395.474 | 100 | CCOC(=O)C=1C=CSC1NC(=O)CNC(C=2C=CC=CC2)C=3C=CC=CN3 |  |  |  |
| C56 | Z118712240 | C20H22N2O4 | 354.399 | 95 | CCN1C=C(C(=O)NC=2C=C(OC)C(OC)=C(OC)C2)C=3C=CC=CC13 |  |  |  |
| C57 | Z53195724 | C21H19N5O2 | 373.407 | 95 | O=C1CCCC2=C1C(C=3C=CC=CC3OCC=4C=CC=CC4)N5N=NN=C5N2 |  |  |  |
| C58 | Z229339834 | C19H24N6O2 | 368.432 | 91 | COC=1C=C(CCC2CCN(CC2)C=3C=CC4=NN=NN4N3)C=C(OC)C1 |  |  |  |
| C59 | Z108853906 | C23H21N5O | 383.445 | 100 | O=C(NC(C=1C=CC=CC1)C=2C=CC=CC2)C(CC=3C=CC=CC3)N4C=NN=N4 |  |  |  |
| C60 | Z195664472 | C21H23N3O3 | 365.425 | 99 | COC=1C=CC(CCNC(C(=O)NC=2C=C(C)ON2)C=3C=CC=CC3)=CC1 |  |  |  |
| C61 | Z27654017 | C22H20N2O2 | 344.406 | 100 | CC(=O)NC=1C=CC(NC(=O)C(C=2C=CC=CC2)C=3C=CC=CC3)=CC1 |  |  |  |
| C62 | Z18520264 | C14H24N4O2S | 312.430 | 100 | CC(C)C1CCC(C)CC1OC(=O)CSC2=NN=NN2C |  |  |  |
| C63 | Z432733912 | C19H17N3O | 303.357 | 97 | O=C(NC=1C=CC=C2C=NC=CC12)N3CCC3C=4C=CC=CC4 |  |  |  |
| C64 | Z736530444 | C16H14ClN3O2S | 347.819 | 100 | ClC=1N=C2C=CC=CN2C1S(=O)(=O)NC3CCC=4C=CC=CC34 |  |  |  |
| C65 | Z971175648 | C13H13N5O2S | 303.339 | 93 | CC1=CC=NN1C=2C=CC=C(NS(=O)(=O)C=3C=NNC3)C2 |  |  |  |
| C66 | Z650466608 | C22H21N3O2 | 359.421 | 99 | O=C(CCC=1C=CN=CC1)NCC=2C=CC=C(C2)C(=O)NC=3C=CC=CC3 |  |  |  |
| C67 | Z435060562 | C20H23N3O4 | 369.414 | 100 | CC(C)C(=O)NC=1C=CC(C)=C(NC(=O)NCC=2C=CC=3OCOC3C2)C1 |  |  |  |
| C68 | Z643123666 | C20H23N3O4 | 369.414 | 95 | COC(=O)C=1C=CC(NCC2(CCCC2)C=3C=CC=4OCCOC4C3)=NN1 |  |  |  |
| C69 | Z454392778 | C20H18N2O3S2 | 398.498 | 96 | CNS(=O)(=O)C=1C=CC(C2=CC=CS2)=C(C1)C(=O)N3CCC=4C=CC=CC34 |  |  |  |
| C70 | Z729771054 | C23H24ClN3O | 393.909 | 98 | Cl.COC=1C=CC=2C=CC=CC2C1CNCC=3C=CC=CC3CN4C=CN=C4 |  |  |  |
| C71 | Z194689474 | C15H12N4OS3 | 360.476 | 95 | C(CSC=1N=CN=C2C=CSC12)CC3=NC(=NO3)C4=CC=CS4 |  |  |  |
| C72 | Z744593102 | C13H10INO3S | 387.192 | 98 | CC=1C=C(NC(=O)C=2C=CC=CC2I)SC1C(=O)O |  |  |  |
| C73 | Z19674372 | C19H16N2O4S | 368.406 | 97 | CC(OC(=O)C=1C=CC(O)=CC1)C(=O)NC2=NC(=CS2)C=3C=CC=CC3 |  |  |  |
| C74 | Z278198642 | C20H24N4O3 | 368.429 | 92 | CC(C)(C)C=1C=CC(OCC(O)CN2N=NN(C=3C=CC=CC3)C2=O)=CC1 |  |  |  |
| C75 | Z114094422 | C19H15N3O3 | 333.340 | 97 | O=C(OCCOC=1C=CC(C#N)=CC1)C=2C=C(NN2)C=3C=CC=CC3 |  |  |  |
| C76 | Z70997953 | C24H19N3O3 | 397.425 | 98 | CC=1C=CC2=NC(=C(NC(=O)C3CC=4C=CC=CC4C(=O)O3)N2C1)C=5C=CC=CC5 |  |  |  |
| C77 | Z244248008 | C23H21N3O3 | 387.431 | 100 | CC1=CC=CN2C=C(COC=3C=CC=CC3C(=O)NOCC=4C=CC=CC4)N=C12 |  |  |  |
| C78 | Z117598840 | C22H23N3O2S | 393.501 | 95 | CN(C)CC(NC(=O)C=1C=CC=C(NC(=O)C2=CC=CS2)C1)C=3C=CC=CC3 |  |  |  |
| C79 | Z195760046 | C18H22N2O | 282.380 | 100 | CCCC(NCC=1C=CC(=CC1)C(=O)N)C=2C=CC=CC2 |  |  |  |
| C80 | Z25939025 | C23H22N4O2 | 386.446 | 95 | O=C(CON1N=NC=2C=CC=CC12)NCCC(C=3C=CC=CC3)C=4C=CC=CC4 |  |  |  |
| C81 | Z52058186 | C21H19N3O2S | 377.459 | 98 | CC1=NC(=CS1)C=2C=CC=C(NC(=O)C=3C=CC(=CC3)N4CCCC4=O)C2 |  |  |  |
| C82 | Z199510658 | C17H21NO4S | 335.417 | 100 | CCOC=1C=CC(=CC1S(=O)(=O)NC=2C=CC=CC2O)C(C)C |  |  |  |
| C83 | Z57705742 | C24H23NO3 | 373.444 | 100 | O=C(CN(CC=1C=CC=CC1)CC=2C=CC=CC2)OCC(=O)C=3C=CC=CC3 |  |  |  |
| C84 | Z55868904 | C19H19N3O2S | 353.438 | 92 | CC=1SC=2N(CN(CC=3C=CC=NC3)CC2C1C)C(=O)C4=CC=CO4 |  |  |  |
| C85 | Z25397974 | C16H15N3OS2 | 329.439 | 100 | O=C(CCCSC1=NN=C(N1)C=2C=CC=CC2)C3=CC=CS3 |  |  |  |
| C86 | Z218663160 | C20H16N4O2S | 376.431 | 100 | NC(=O)C(SC1=NN=C(C2=CC=CO2)N1C=3C=CC=CC3)C=4C=CC=CC4 |  |  |  |
| C87 | Z106784726 | C22H23N5S | 389.516 | 92 | CC(C)C=1C=CC(=CC1)C(NCC2=NN=NN2C=3C=CC=CC3)C4=CC=CS4 |  |  |  |
| C88 | Z88331415 | C18H14N2O2S | 322.380 | 95 | CC(=O)C1=CC=C(S1)C=2C=CC=CC2NC(=O)C=3C=CC=CN3 |  |  |  |
| C89 | Z135439958 | C21H21N3O3 | 363.409 | 97 | CCN(CC)C(=O)C=1C=CC=C(NC(=O)C2=CC(=O)NC=3C=CC=CC23)C1 |  |  |  |
| C90 | Z85917655 | C19H18N4O2S | 366.436 | 100 | CC1=C(C(C2=CC=C(C)S2)N3N=CN=C3N1)C(=O)OCC=4C=CC=CC4 |  |  |  |
| C91 | Z19883124 | C20H19N3O3S | 381.448 | 100 | COC=1C=CC(=CC1)N2C(SC3CCOC3=O)=NN=C2C=4C=CC=C(C)C4 |  |  |  |
| C92 | Z27798110 | C19H18N2O2 | 306.358 | 100 | CC=1ON=C(C1C(=O)NC=2C=CC=C(C)C2C)C=3C=CC=CC3 |  |  |  |
| C93 | Z185185866 | C19H21N3O2S | 355.453 | 99 | CC=1C=CC(C)=C(C1)S(=O)(=O)NC(C2=NC=CN2C)C=3C=CC=CC3 |  |  |  |
| C94 | Z57003550 | C18H16N2O4S | 356.395 | 100 | OC(=O)CCC1=CC=C(C2=CC=CS2)N1NC(=O)C=3C=CC(O)=CC3 |  |  |  |
| C95 | Z26545746 | C22H17N3O2 | 355.389 | 91 | CN1N=C(C(=O)NC=2C=CC=CC2C=3C=CC=CC3)C=4C=CC=CC4C1=O |  |  |  |
| C96 | Z146826758 | C22H22N4O2 | 374.435 | 100 | CC1=NN(C(C)=C1C(=O)NC=2C=CC=C(C2)C(=O)NC3CC3)C=4C=CC=CC4 |  |  |  |

*****Note the order of the compounds shown is not related to their predicted affinity from the virtual screening.
