# Supplementary material for: AI-based discovery and cryoEM structural elucidation of a KATP channel pharmacochaperone
Source: eLife. 2025 Mar 26;13:RP103159. doi: 10.7554/eLife.103159 (PMC11942174; doi:10.7554/eLife.103159)
Supplement: Supplementary file 2. [file elife-103159-supp2.docx]

**Supplementary File 2. CryoEM and model statistics of the Aekatperone-bound SUR1/Kir6.2 structure**

| **Data Collection** |  |
| --- | --- |
| Microscope | Titan Krios |
| Voltage (kV) | 300 |
| Camera | Gatan K3 |
| Camera mode | Super-resolution |
| Defocus range (µm) | -1.0 ~ -2.5 |
| Movies | 21,012 |
| Frames/movie | 74 |
| Exposure time (s) | 3.2 |
| Frame rate (/s) | 23 |
| Magnified pixel size (Å)* | 1.0515 |
| Total dose (e^-^/Å^2^) | ~55 |
|  |  |
| **Reconstruction** |  |
| Software | Cryosparc 4.2.1 |
| Symmetry | C1 |
| Mask | 1xSUR1 plus 4x Kir6.2 |
| Particles refined | 125,416 C4 symmetry-expanded particles |
| Resolution (masked) | 4.1 Å |
|  |  |
| **Refinement Statistics** |  |
| PDB ID^1^ | 9DFX |
| EMDataResource | EMD-46820 |
| Atoms | 20087 |
| Protein residues | 2781 |
| Map CC (masked) | 0.76 |
| FSC model (0.143, 0.5) | 4.1, 5.9 |
| Clash score | 5.39 |
| Molprobity score | 1.30 |
| Cβ deviations | 0 |
| Rotamer outliers (%) | 1.04 |
| ADP (mean protein) | 283.52 |
| ADP (mean ligands) | 293.98 |
|  |  |
| **Ramachandran** |  |
| Outliers | 0.00 |
| Allowed | 1.81 |
| Favored | 98.19 |
|  |  |
| **Bonds (RMSD)** |  |
| Length (Å) | 0.001 |
| Bond angles | 0.372 |
|  |  |

^1^PDB ID 9DFX & EMD-46820 contains the Kir6.2 tetramer and one of four SUR1 subunits of the K_ATP_ channel

*Super-resolution pixel size 0.52575 Å
